# Compatibilizer Efficiency in Enhancing Marine Plastic Waste Valorization Through Simulated Recycled Plastic Blends

**DOI:** 10.3390/polym16233441

**Published:** 2024-12-08

**Authors:** Sibele Piedade Cestari, Pedro Veiga Rodrigues, Ana Cristina Ribeiro, Maria Cidália Rodrigues Castro, Vasco Cruz, Ana Rita Torres, Nuno Ramos, Ana Vera Machado

**Affiliations:** Department of Polymer Engineering, Institute for Polymers and Composites (IPC), Campus de Azurém, University of Minho, 4804-533 Guimarães, Portugal; sibele.cestari@piep.pt (S.P.C.); pedro.rodrigues@dep.uminho.pt (P.V.R.); anacmr05@gmail.com (A.C.R.); cidaliacastro@dep.uminho.pt (M.C.R.C.); vasco.cruz@dep.uminho.pt (V.C.); ana.torres@dep.uminho.pt (A.R.T.); nuno.ramos@dep.uminho.pt (N.R.)

**Keywords:** recycling, marine environment plastics, polymer blends, blend properties

## Abstract

This study investigated the optimal combination of compatibilizers and stabilizers to enhance the value of marine environment plastic (MEP). The composition of the plastics was analysed, and a simulated recycled plastic blend (sMEP) was prepared based on a simplified composition of actual MEP. Different concentrations of three commercial compatibilizers (C1, C2 and C3) were tested to improve tensile strength. The tensile tests indicated that the blend compatibilized with 10 wt.% C3 (polypropylene grafted with maleic anhydride) exhibited the highest increase in tensile strength. This optimal compatibilization was then combined with two commercial stabilizers and applied to a simulated MEP blend. Scanning electron microscopy images showed that all blends had a continuous polyethylene phase with dispersed poly(ethylene terephthalate) (PET) and polypropylene (PP) droplets. The simulated blend with 10 wt.% C3 exhibited a reduced PET droplet size in the dispersed phase. Differential scanning calorimetry results revealed a decrease in polyethylene crystallinity and an increase in PP crystallinity. The improved properties of the blend were attributed to the effectiveness of the C3 compatibilizer in enhancing the interface between the PP and PET phases. An effective formulation was developed to valorise marine-sourced plastics by leveraging existing scientific knowledge and accessible commercial additives. Applying this enhanced formulation to real MEP not only demonstrated its effectiveness, but also highlighted a practical approach for reducing plastic pollution and supporting circular economy principles, contributing to environmental conservation efforts.

## 1. Introduction

The most recent research initiatives focus on the sustainability issue and the role of plastic waste within it. This has resulted in significant funding being allocated to specific topics related to this theme [1,2]. However, instead of addressing the underlying cause of the problem, much of the research primarily focuses on treating the symptoms, particularly the presence of plastic in the environment. The irresponsible disposal of plastic by society is what ultimately leads to the pollution of the environment and harm to living beings. Scientific studies have shown that the issue lies not with plastic as a material itself. When it comes to marine environments, the damage occurs because plastic and other items are in places where they should not be [3]. Recent estimates indicate that nearly 70% of all produced plastic waste (more than 252 million tonnes in 2021) is not treated and continues to end up in the environment with negative impacts [4]. The packaging industry is undoubtedly responsible for 50%, followed by the construction, industrial, and agricultural sectors at 30%. To address this issue, several strategies have been adopted to reduce plastic pollution’s effect on the environment. Recovery and mechanical recycling have been used for several years, often focusing on downcycling due to the reduced properties of the resultant plastic compared to the original material, making it unsuitable for food contact applications. Conversely, research has developed alternative polymers with biodegradable capabilities to lessen environmental harm [5]. Upcycling strategies have been employed to provide high-value solutions, such as improved plastic properties, pure H2, carbon-based nanomaterials and depolymerization, among others [6]. The attention surrounding polymer waste found on beaches is mainly due to its visibility. In reality, the number of animals entangled in plastic nets has decreased since 2000 [7]. Even the well-known Pacific Garbage Patch has been found to be different from what was initially believed. Contrary to common claims, it cannot be seen from space and mainly consists of fishing industry nets (46%) and debris from the Japanese tsunami of 2011 (20%) [8]. Interestingly, it has been discovered that construction materials and cigarette butts/filters make up the majority of beach litter in Europe [9], and the responsibility for the generation of anthropogenic litter on beaches primarily lies with beach users [10]. However, no one has called for a ban on building materials, smoking or using beaches. Under environmental conditions, polymers can undergo several degradation mechanisms, including physical (mechanical forces, such as abrasion), chemical (photochemical, thermos-oxidative, and hydrolytic degradation) and biological (microorganisms) degradation [11]. In all cases, the integrity of the polymer’s chemical structure is disrupted. The main degradation mechanism of polyethylene and polypropylene is oxidative reactions, initiated by exposure to UV radiation or heat, leading to chain scission and the formation of smaller hydrocarbons and carbonyl groups [12,13]. Additionally, poly(ethylene terephthalate) is highly susceptible to hydrolysis and photo-oxidation, resulting in the cleavage of its ester bonds [14]. From these degradation mechanisms, smaller plastic particles with a lower molecular weight (shorter polymeric chains) are formed, leading to the emergence of microplastics. Compared to neat polymer, degraded plastic exhibits lower properties, affecting its application and valorisation.

According to Galgani (2015), plastics are often the main component of marine litter, sometimes comprising all of the floating waste. Studies have shown that the density of debris on beaches is around 1 item per square meter [15]. Data from the Convention for the Protection of the Marine Environment of the North-East Atlantic (OSPAR) in 2022 indicated an upward trend in marine litter on beaches in North Portugal. The most common types of litter found on beaches in the Northeast Atlantic marine region are fishing nets, large and small pieces of polystyrene, caps and lids [16]. However, collecting and sorting post-consumer plastic waste from any environment is challenging and expensive. Therefore, the most efficient recycling method for plastics in this marine environment skips the sorting process, mixes all the materials, and produces a mechanically recycled polymer blend [17]. The “Evaluation and valorisation of plastics and microplastics in marine environment—MarPlas” project found that the plastic waste on beaches in North Portugal mainly consists of polyolefins and poly(ethylene terephthalate) from post-consumer plastics such as bottles, bags, and caps/lids. When these materials are mixed together, they create an incompatible blend with weak interfacial bonds and poor mechanical and physical properties [18].

The use of compatibilizers is widespread in polymer recycling; they can address contaminants in immiscible polymer elements when they exceed achievable thermodynamic miscibility or compatibility limits. The compatibilization process can be achieved by modifying the predominant polymer to provide reactive sites for covalent bonding with minor polymer elements. New synthetic approaches have produced highly effective block copolymer compatibilizers that incorporate the morphological characteristics of the polymers present in the blend [19]. In general, compatibilizers based on polyethylene or polypropylene grafted with maleic anhydride (PE-g-MA and PP-g-MA, respectively), styrene–ethylene-co-butene–styrene (SEBS), ethylene–vinyl acetate (EVA) and ethylene–methacrylic acid copolymer (EMA) are employed in the compatibilization of poly(ethylene terephthalate) (PET)/polyolefins [20,21,22,23,24]. Fasce and co-workers studied PET/PE 50/50 blends compatibilized with varying amounts of EMA. They reported increased adhesion at the interface with 7 wt. % EMA, attributing the overall improvement in mechanical properties to fibrillation in the PET phase [25]. Nomura and co-workers synthesized PET/PE multiblock copolymers to compatibilize a PET/PE 80/20 blend. They observed a reduction in PE droplet size and an increase in strain at break with the addition of 0.5 wt.% compatibilizer, which they linked to the localization of the multiblock copolymer at the blend’s interface [26]. Tang et al. tested three commercial compatibilizers based on ethylene acrylate copolymer (ELVALOY™ AC 2016 Acrylate Copolymer (EAA), ELVALOY™ PTW Copolymer (PTW), and SURLYN™ 1802 Ionomer (Surlyn)) in a 50/50 PET/HDPE blend [27]. They concluded that EAA did not improve the interface or mechanical properties of the blend, but PTW and Surlyn enhanced elongation at break and toughness. These authors emphasized the importance of understanding the molecular arrangement of the blend by considering the combination of mechanical properties, crystallinity and morphology data, as well as estimating the location (interface vs. bulk) of the compatibilizer molecules.

Stabilisers have been essential for protecting and extending the lifespan of recovered polymers since the late 1980s [28]. Polyolefin degradation during processing is well understood and documented in the literature. To limit the radical chain reactions occurring during the production and use of the polymer, stabilising chemicals (antioxidants) must be added to polyolefins [29]. These processes, known as autoxidation reactions, begin with the production of free radicals through light exposure, heat, or shear. They then progress to a series of autocatalytic radical chain reactions that result in the formation of peroxy-, hydroperoxy-, alkoxy- and hydroxyl-radicals, as well as additional carbon-centred radicals through hydrogen abstraction mechanisms. Radical chain reactions are mitigated by phosphite and phenolic antioxidants working together. Phosphites inactivate hydroperoxides, while phenolic antioxidants inactivate oxygen-cantered radicals. The processing and stability of the material can be enhanced by using UV absorbers and other stabilisers. The amounts of active species (such as phosphite) present in recyclates are often insufficient to protect the material from degradation during reprocessing. Contaminants and impurities affect stability and the choice of stabilisation technique for the polymers. It is expected that recycling materials, such as marine plastic waste, which has been subjected to extreme environmental deterioration and contamination, will require the addition of one or more stabilisers.

When collected from the environment, plastic material already exhibits some degree of degradation, which are unsuitable to be blended with virgin polymer. Therefore, this study investigates the optimal combination of compatibilizers and stabilizers to valorise marine environment plastic (MEP), searching for new strategies to upcycle the marine plastic waste collected. Thus, first the composition of the collected MEP was assessed, and then a recycled plastic blend was prepared to simulate this combination. Various amounts of commercial compatibilizers were tested to achieve the best improvement in mechanical properties. The optimal compatibilization was achieved using two commercial additives, and the enhanced formulation could be applied to the real MEP.

## 2. Materials and Methods

The MEP was collected at the sandbank and mouth of the Cávado River in Esposende city (Braga, Portugal) in 2022. The recycled PE and PP were generously donated by Recuplás—Reciclagem de Plásticos and R3Natura (Braga, Portugal). The recycled PET was donated by Ecoibéria (Braga, Portugal). The compatibilizers studied were as follows: polyethylene grafted with maleic anhydride (C1) Fusabond^®^ E226 (supplied by Dow Chemical, Aveiro, Portugal), containing 0.5–1 wt.% of grafted maleic anhydride; styrene–butadiene block copolymer (C2) Styroflex^®^ 2G66 (donated by INEOS Styrolution), Barcelona, Spain, a thermoplastic elastomer with a hard–soft–hard block sequence; and polypropylene grafted with maleic anhydride (C3) Polybond^®^ 3200 (donated by AddivantTM, Basel, Switzerland), containing 0.8–1.2 wt.% of grafted maleic anhydride. The stabilisers were IrgaCycleTM PS 030 (S1), which improves the durability of rigid articles made with polyolefin recyclates, and IrgaCycleTM XT 034 (S2), for polyolefin recyclates with significant levels of impurities, fillers, or pigments, both donated by Colorstar/BASF-SE (Porto, Portugal).

### 2.1. MEP Preparation

About 4 kg of marine environment plastics (MEPs) were hand-washed with neutral soap, rinsed with running water and dried overnight in an oven at 60 °C. The sand from the washed and dried MEPs was measured. We removed the labels, retaining the plastic ones and discarding the paper labels. After separation and identification, the films were pressed in a heated press and hand-cut. All materials (solids and compressed films) were ground in a knife mill to a flake size of approximately 5 mm.

### 2.2. Blend and Specimen Preparation

Since the MEP amount was small, a simulated MEP (sMEP) of recycled plastics was prepared based on the simplified composition of the real MEP: 42 wt.% HDPE, 13 wt.% LDPE, 25 wt.% PET, and 20 wt.% PP. About 2 kg of sMEP were processed as received (chunks and flakes) in a Leistritz extruder model LSM co-rotating twin-screw configuration, with a temperature profile of 180 °C (feeding zone)/240 °C/240 °C/240 °C/240 °C/250 °C/260 °C (die) at 100 rpm. The compatibilizers and stabilizers were cryogenically ground in a Retsch mill model ZM 100 and added in three different contents (2, 5 and 10 wt.%) to the sMEP (Table 1).

The sMEP blends were compression-moulded into laminates with a thickness of approximately 0.7 mm in a heated press at 270 °C, 7.7 MPa, for 5 min, and then cooled in a press at 25 °C, 4.8 MPa, for 5 min. After characterisation, to the blend that exhibited best tensile resistance (sMEP10 wt.% C3) was added with 0.5 wt.% stabiliser S1, 2 wt.% stabiliser S2 and a combination of both stabilisers (0.5 wt.% S1 + 2 wt.% S2).

### 2.3. Characterisation

#### 2.3.1. Density

The density tests were performed according to ASTM D-792 [30], in isopropyl alcohol at 25 °C (ρ = 785 kg·m^−3^), using five specimens of each sample.

#### 2.3.2. Mechanical Properties

Mechanical tests were performed in Zwick/Roell universal testing machine Z005 model (Ulm, Germany), using a load cell of 5 kN. The tests of sMEP were conducted according to the ASTM D882 [31], test speed of 5 mm/min and specimens of 100 × 10 × 0.7 mm. The percentage variation of Young’s modulus was determined, as the secant modulus at 2% of deformation. The results consider the mean of the five specimens for each sample.

#### 2.3.3. Morphology Spetroscopy

Morphological analysis was performed using ultra-high resolution field emission gun scanning electron microscopy (FEG-SEM) and a NOVA 200 Nano SEM (FEI, Amsterdam, Netherlands). Samples were fractured in liquid nitrogen and coated with a thin film (2 nm) of Au-Pd (80–20 wt. %), using a high-resolution sputter coater (208HR Cressington Company, Watford, UK). To evaluate the dispersion state of the different polymers in the incompatible blend, the PET droplet size was assessed by adapting and simplifying the methods of Novais, Jamali, and Carson. As it was not possible to discern the PET phase from the PP phase in SEM, the samples sMEP, sMEP2 C3, sMEP5 C3, and sMEP10 wt.% C3 were extracted with hexafluoroisopropanol (HFIP) at room temperature to remove the PET phase, and then analysed in two different regions using SEM images at 1000× magnification. The area of the holes with a maximum dimension of at least 5 µm was assessed using ImageJ software (available at https://imagej.net/, accessed on 1 October 2024).

#### 2.3.4. Fourier Transform Infrared Spectroscopy (FTIR)

The spectra were acquired at room temperature in an FTIR 4100 Jasco spectrometer apparatus (Tokyo, Japan), in attenuated total reflectance (ATR) mode, between 4000 and 600 cm^−1^ wavelength range, using 64 scans.min^−1^ and 8 cm^−1^ resolutions. Thin films of all samples were prepared by compression moulding in a hot press at 270 °C under a pressure of 7.7 MPa. To evaluate the effect of the compatibilizers in the scission of the ester bond of rPET chains, the ratio between the absorption of both the variable carbonyl band at 1716 cm^−1^ and the invariable methylene band at 2914 cm^−1^ was determined; this rate was called the carbonyl index (CI) [32,33].

#### 2.3.5. Differential Scanning Calorimetry (DSC)

The samples were analysed using Netzsch DSC 200 F3 Maya equipment (Selb, Germany) under a nitrogen atmosphere, following ASTM D3418-A [34]. In the first cycle, the sample was heated from 30 to 270 °C at a rate of 10 °C·min^−1^, and held at 270 °C for 1 min. The second cycle involved cooling at a rate of 10 °C·min^−1^ until reaching 30 °C. In the third cycle, the same temperature range and heating rate as the first cycle were applied. The crystallization temperature (T_c_) was determined from the second cycle, while the polymer crystalline melting temperature (Tm) and degree of crystallinity (*X_c_*) were obtained from the third cycle. The *X_c_* was calculated according to Equation (1):

(1) $$X_{cPolymer}=\frac{H_{mPolymer}}{H_{mPolymer}^{0}}\;\cdot af,$$

where $H_{m}$ is the melting enthalpy of the polymer, $H_{m}^{0}$ is the theoretical melting enthalpy of 100% crystalline polymer (293 J·g^−1^ for PE, 209 J·g^−1^ for PP and 140 J·g^−1^ for PET) and *af* is the adjustment factor (Table 1) adopted to estimate the polymer percentage being melted in a given temperature range based on the percentage of polymer type present in the blend [35,36,37]. As C1 is PE and C3 is PP, they were added to their polymer type in the *af.*

## 3. Results and Discussion

### 3.1. MEP Composition

The quantitative and qualitative evaluation MEP characterisation is listed in Table 2, which also presents the amounts and percentages of solids and films. The MEP artefacts were categorised by solids, films and polymer type, based on their conventional identification symbols. Among the unidentified objects, some had no symbol (e.g., straws, bottle caps, bags) and were identified using information from public sources or literature. Others (mono- and multilayer films, labels, ropes, fishing nets, and strings) were identified through FTIR analysis. A coffee pod, a syringe plunger and a toy could only be identified through density and DSC tests. The density/T_m_ results were: 900 kg·cm^−3^/161 °C for the coffee pod, 950 kg·m^−3^/126 °C for the syringe plunger, and 0.92 g/cm^3^/166 °C for the toy. According to the literature, the density and Tm obtained for the samples are consistent with results found for PP (coffee pod), LDPE (syringe) and PP (toy) [38,39,40,41,42]. From the quantitative analysis, the MEP sample was mainly composed of polyolefins (48 wt.% PE and 17 wt.% PP) and polyesters (21 wt.% PET), which was used as a basis to produce the simulated MEP (sMEP). Most of the collected samples were derived from packaging products, like films and bottles for food packaging.

### 3.2. Tensile Properties

**Figure 1:**
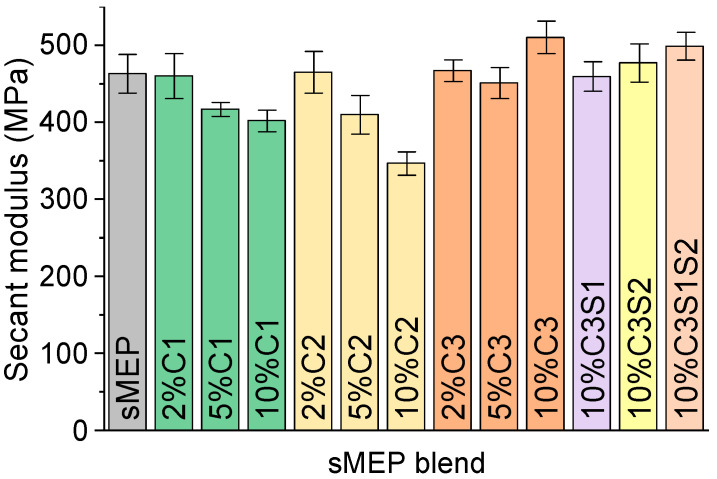
Secant moduli of the sMEP blends.

As the sMEP blends’ tensile curves did not exhibit a linear region (Figures S1 and S2), the secant moduli of each blend were calculated (Figure 1). The only blend showing an increase in tensile strength (10% ± 0.21) was the one compatibilized with 10 wt.% C3. Since the study aimed to add value to the recycled plastic, the focus was solely on C3-compatibilized blends. The S12 combination in the sMEP had the smallest loss in tensile strength, at −7%. This result aligns with the findings of Ahmadlouydarab and colleagues, who observed similar behaviour in PP/PET blends compatibilized with PP-g-MA [18].

### 3.3. Morphology

**Figure 2:**
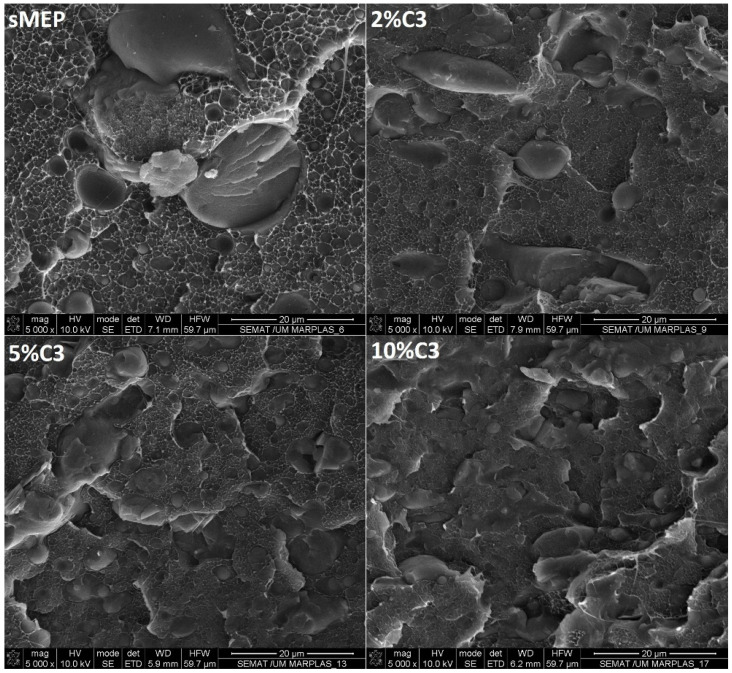
SEM images of sMEP, sMEP2C3, sMEP5C3 and sMEP10 wt.%C3 (×5000).

All blends exhibited a continuous PE phase with dispersed PET and PP droplets. Figure 2 compares the images of sMEP and the C3-compatibilized blends magnified 5000×. The heterogeneity of the fractured section appears to decrease with increasing C3 content. Regions and large droplets of the dispersed phase tend to disappear from the 5 wt.% C3 content onward. The PP and PET dispersed phases are not easily distinguishable in these images. After extracting the PET phase, the dispersed phases of the sMEP could be accurately assessed.

**Figure 3:**
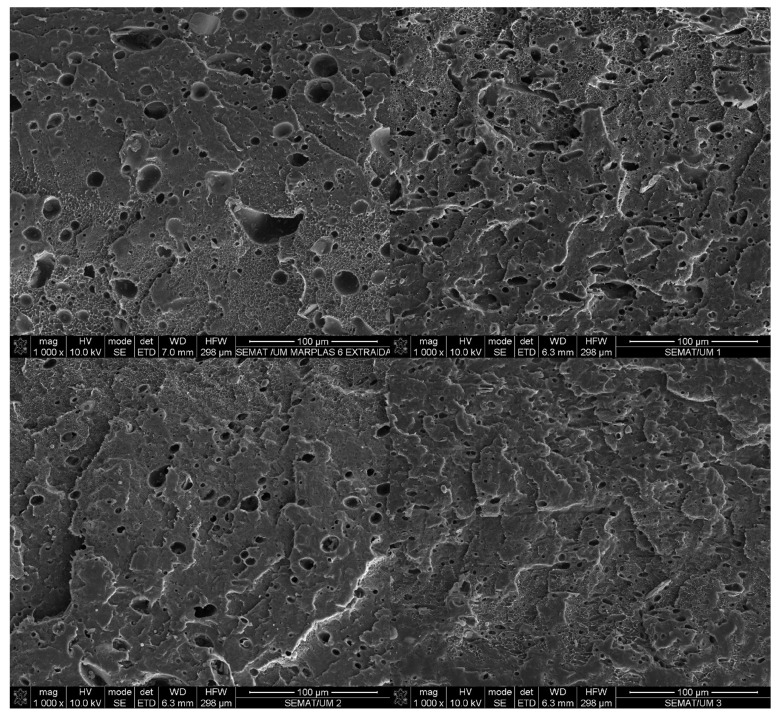
SEM images of PET-extracted sMEP, sMEP2C3, sMEP5C3 and sMEP10 wt.% C3 (×1000).

**Figure 4:**
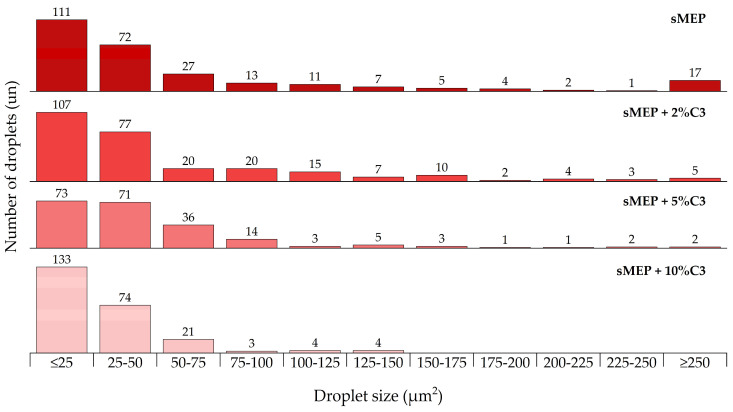
Histogram of PET droplet size distribution by area range.

**Figure 5:**
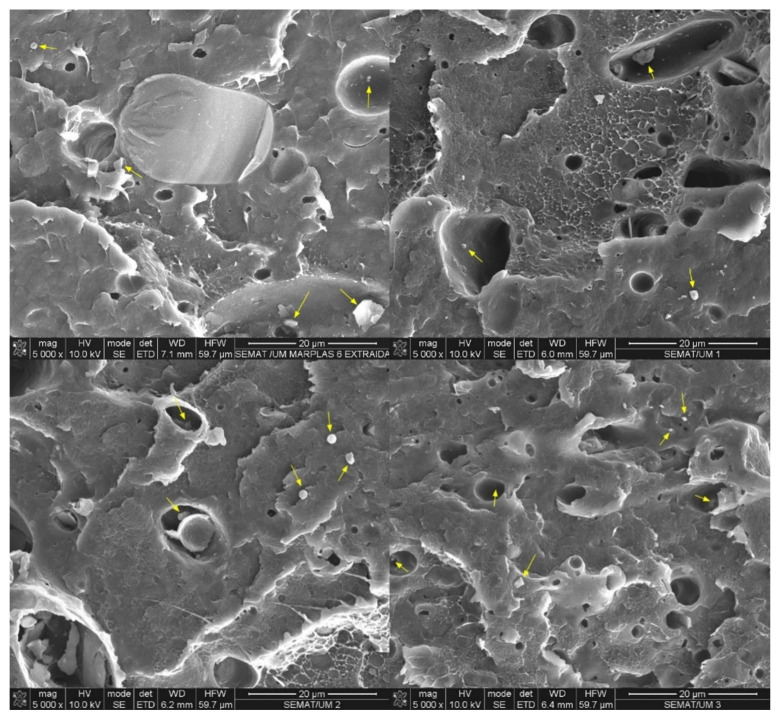
SEM images of PET-extracted sMEP, sMEP2C3, sMEP5C3 and sMEP10 wt.%C3 showing PP crystals formed at the interface of PE/PET (×5000). Yellow arrows show PP crystals developed at the interface between PE and PET.

The SEM images of the extracted samples (Figure 3) showed a decrease in PET droplet size as the C3 content increased. These smaller droplets provided a better interface between the PET fraction and the polyolefin of the blend. The droplet size results were presented as the average droplet size per material. The uncompatibilized blend showed an average droplet area of 70 µm^2^, while the 2 wt.% C3 had 64 µm^2^, the 5 wt.% C3 had 48 µm^2^, and the 10 wt.% C3 showed the smallest droplet size of 32 µm^2^, equivalent to a 54% reduction in the average droplet size of the PET dispersed phase. A histogram was prepared to show the distribution of droplet sizes by area range (Figure 4, Table 3). Areas above 200 µm^2^ correspond more to regions than droplets and tend to disappear in the 10 wt.% C3 blend. In the ×5000 magnification images (Figure 5), PP crystals formed at the interface between PE and PET can be observed. The sMEP exhibited a partial wetting morphology regarding the phase size and shape [43].

**Figure 6:**
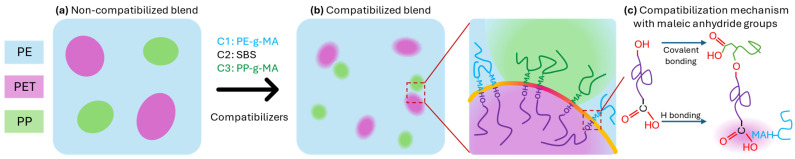
Schematic representation of the compatibilization mechanism of the PE/PET/PP blend: (**a**) non-compatibilized blend, (**b**) compatibilized blend (the line with a colour gradient at the interface represents the interaction between each pair of phases, PE/PET and PP/PET, linked by the compatibilizer), and (**c**) reaction between MA groups and PET.

The compatibilization mechanism is illustrated in Figure 6, adding compatibilizers to immiscible blends decreases the interfacial tension between incompatible polymers. Due to the non-polar nature of polyolefins (PE and PP) and the polar nature of PET, it is crucial to select the appropriate compatibilizer for each system. Thus, compatibilizers with reactive functional groups, such as maleic anhydride (MA), are preferred to connect the polar (PET) and non-polar (PE and PP) phases. As depicted in Figure 6, the hydroxyl end groups of PET react with MA groups from PE-g-MA and PP-g-MA compatibilizers through covalent bonding [44,45]. Additionally, hydrolysed MA (MAH) groups can interact with the ester groups of PET through hydrogen bonding, enhancing the compatibilizing effect [46]. For the C2 compatibilizer, the manufacturer states that this SBS copolymer is more polar than regular SBS but does not specify the type of modification. The primary compatibilization mechanism is likely hydrogen bonding. As shown in tensile analysis and SEM, C3 proves to be the most effective compatibilizer for reducing interfacial tension in the blend and improving the interaction between the PP and PET phases. This strategy decreases particle sizes, reducing the heterogeneity of the blend. These results are in agreement with ones published in the literature on the compatibilization of polymers blends of PE/PET [25,26].

### 3.4. FTIR

**Figure 7:**
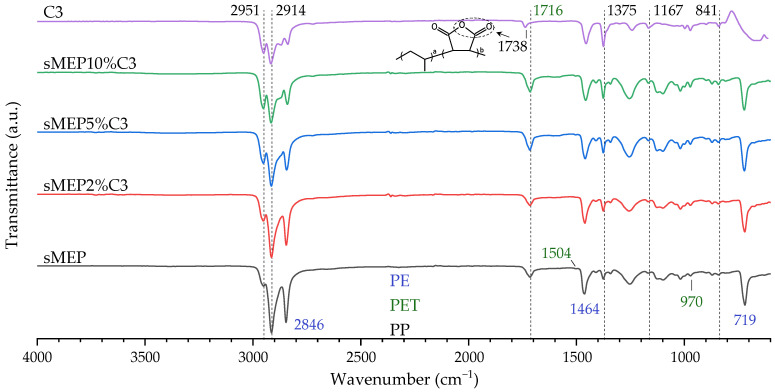
FTIR spectra of sMEP, sMEP2%C3, sMEP5%C3 and sMEP10%C3.

Figure 7 shows the FTIR spectra of the sMEP, C3, and C3-compatibilized blends. The pure blend spectra displayed characteristic HDPE peaks at 2914 cm⁻^1^ and 2846 cm⁻^1^, attributed to the asymmetric and symmetric stretching vibrations of methylene (–CH_2_–) groups; 1464 cm⁻^1^ for bending deformation of methylene groups; and 719 cm⁻^1^ for CH_2_ rocking deformation of methylene groups of PE [47,48]. Additionally, the characteristic PET bands appeared at 1716 cm⁻^1^, attributed to the stretching vibration of the C=O group; at 970 cm⁻^1^ for the methylene of the glycol ethylene linkage (related to the trans conformation of the H_2_C–O group); and a weak band at 1504 cm⁻^1^ for the C=C bond of the rPET aromatic ring [27,32,49]. The PP bands were observed at 2951 cm⁻^1^ for CH_3_ asymmetric C–H stretching; at 2914 cm⁻^1^ for CH_2_ asymmetric C–H stretching; at 1375 cm⁻^1^ for CH_3_ symmetric bending; at 1167 cm⁻^1^ for methyl group wagging vibration; and at 841 cm⁻^1^ for CH_2_ rocking vibration [32,48,50,51]. In the C3 spectrum, the band at 1738 cm⁻^1^ was attributed to the characteristic anhydride group of maleic anhydride [47]. The area of the carbonyl band at 1716 cm⁻^1^ increased with the amount of compatibilizer, as seen in Table 4. This increase in CO indicates that the PET fraction of the blend reacted with the maleic anhydride of C3, grafting some PET chains and enhancing the interaction between PP and PET domains, as observed in the SEM images.

### 3.5. Thermal Properties

**Figure 8:**
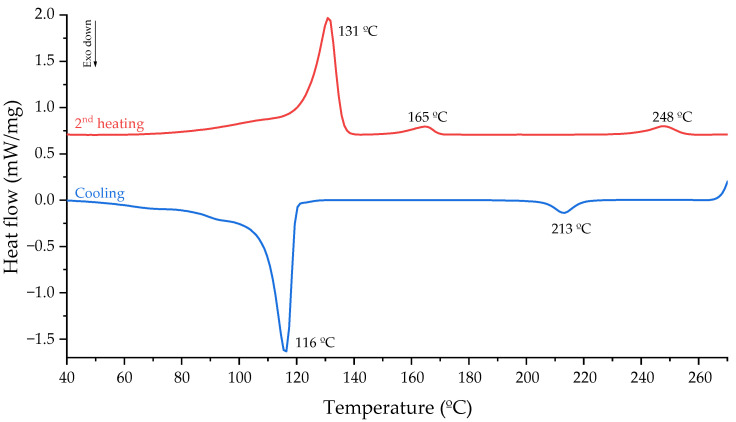
Calorimetric curves of sMEP.

The sMEP DSC curves (Figure 8) show three melting and two crystallization peaks. The first peak occurred around 130 °C (PE region), the second peak around 165 °C (PP region), and the third peak around 250 °C (PET region). The PE peak (131 °C) was slightly below the characteristic melting temperature of high-density polyethylenes. The peak’s shape indicated the heterogeneity of the PE fraction of sMEP (42% HDPE and 13% LDPE). The presence of LDPE’s typical crystalline material of smaller size/perfection was noted, melting between 60 and 100 °C and obscuring PET’s Tg. The decrease in the PE portion’s Tm can be attributed to two factors. First, steric hindrance restricted crystal growth, leading to a drop in PE crystallinity. Second, the presence of already solidified PP and PET crystals increased melt viscosity, further contributing to the decrease in TmPE. The PP melting peak occurred around 165 °C, and an individualised TcPP was not observed in the cooling curve, suggesting coincident crystallization of PP and PE phases. The PET peak appeared around 248 °C, the typical melting temperature for this polymer.

**Figure 9:**
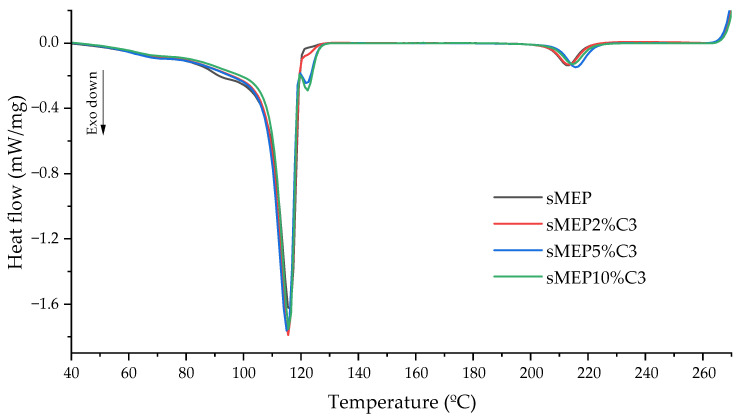
Overlay of the cooling curves of sMEP, sMEP2%C3, sMEP5%C3 and sMEP10%C3.

Table 5 and Figure 9 present the key thermal transitions and the thermographs for all materials, respectively, along with the crystallinity (Xc) values and their changes based on the types and quantities of compatibilizers used. The crystallinity of the PE portion of the sMEP showed a declining trend, with the blend containing 10% C3 exhibiting the most significant decrease (19%). Except for the blends with 5 and 10% of C1 and C2, the PP fraction displayed a rising tendency even in blends with non-PP-based compatibilizers. The sMEP5%C3 and sMEP10%C3 blends showed the largest increases in XcPP (108 and 136%, respectively). No definitive trend in crystallinity variation was observed in the PET portion of the blends. The blends containing 2, 5 and 10 wt.% of C3 showed variations of 3, 18 and −10%, respectively. The decrease in crystallinity could be attributed to the reduction in PET’s crystal and/or chain size, likely caused by increased acidolysis resulting from the highest maleic anhydride content [24,35,37].

With the addition of C3, acidolysed PET chains were grafted onto the compatibilizer’s main structure, forming a copolymer with a backbone soluble in the PP fraction of sMEP. During the crystalline melting of PP, this copolymer could anchor to PET crystals still in the solid state, likely increasing the heat required to complete the PP melting process. Therefore, despite no change in TmPP, an increase in ∆HmPP was noticed.

Initially, the addition of C3 increased the Xc of the PET portion. This increase could be attributed to a better crystalline arrangement of the PET’s crystallizable portions, possibly caused by by-products from the grafting process. Impurities or catalyst residue might have acted as nucleating agents up to a C3 content of 5 wt.%. The overlay of the cooling curves of the blends shows that the TcPET of sMEP5 wt.%C3 occurred at a slightly higher temperature with an enlarged peak area. This observation suggests the formation of smaller, less perfect PET crystals, indicative of the effect of nucleating agents. At 10 wt.% C3 content, there is no longer an increase in the Xc observed, as the TmPET remains the same, but the ∆HmPET decreases back to the value of the uncompatibilized blend.

Regarding the stabilized blends, the crystallinity of the PE part significantly decreased due to the stabilizers. Compared to the sMEP10%C3 blend, the stabilizers S1 and S2 enhanced the XcPP by 17 and 7%, respectively, while the S12 combination reduced it by 16%. S2 had a nucleating effect on PET, resulting in many crystallization centres. This effect led to an increased number of crystals (a 209% increase in XcPET) and smaller crystal sizes, with a melting temperature 10 °C lower than the TmPET of the sMEP10%C3 blend.

## 4. Conclusions

Despite the challenge of working with an incompatible ternary polymer blend, using recycled plastics proved to be even more difficult. The quantitative and qualitative characterization of MEP revealed a mixture of 75% polyolefin and 25% PET, with an insignificant sand content that did not interfere with the average extrusion process. In the simulated blend, reactive extrusion with 10 wt.% C3 resulted in an increase in tensile strength of approximately 10%. The stabilizers decreased the tensile strength of sMEP, as the three options—S1, S2 and S12—interfered with the matrix, causing material rupture. The FTIR results showed that the increase in C3 content raised the carbonyl index, suggesting the grafting of PET chains into the C3 backbone through ester bonds formed after the reaction of carboxyl PET end-groups with the maleic anhydride groups of C3. This reaction improved the interface between the dispersed PP and PET phases, as evidenced by the reduction in PET droplet size.

This research allows us to predict which compatibilizer, reactive or non-reactive, would have the best performance in the composition of a 55% PE, 25% PET and 20% PP blend. An effective formulation was developed to valorise the recycling of marine environment plastics by leveraging existing scientific knowledge and utilizing readily available commercial additives. Ultimately, this study advances strategic methodologies for recycling complex polymer blends, aligning with growing environmental and sustainability goals.

## Figures and Tables

**Table 1:** Composition of the sMEP blends and adjustment factor for degree of crystallinity.

Blend	Weight Percentage (wt.%)						Adjustment Factor		
	sMEP	C1	C2	C3	S1	S2	PE	PP	PET
sMEP	100.0						0.550	0.200	0.250
sMEP2%C1	98.0	2.0					0.559	0.196	0.245
sMEP2%C2	98.0		2.0				0.539	0.196	0.245
sMEP2%C3	98.0			2.0			0.539	0.216	0.245
sMEP5%C1	95.0	5.0					0.550	0.200	0.245
sMEP5%C2	95.0		5.0				0.571	0.190	0.238
sMEP5%C3	95.0			5.0			0.524	0.190	0.238
sMEP10%C1	90.0	10.0					0.524	0.238	0.238
sMEP10%C2	90.0		10.0				0.550	0.200	0.238
sMEP10%C3	90.0			10.0			0.591	0.182	0.227
sMEP10%C3S1	90.5			9.0	0.5		0.500	0.182	0.227
sMEP10%C3S2	89.0			9.0		2.0	0.500	0.273	0.227
sMEP10%C3S12	89.0			9.0	0.5	2.0	0.550	0.200	0.227

**Table 2:** Type and percentage of materials in the MEP sample.

Material	Subtotal Weight		Weight (g)		Total Weight Percentage (wt.%)	
	(g)	(wt.%)	Solids	Films	Solids	Films
PE	1934.3	48.4	1464.5	469.8	48.9	48.4
PET	850.3	21.3	850.3	-	28.4	-
PP	671.7	16.8	351.0	320.7	11.7	33.1
PVC	48.2	1.2	48.2	-	1.6	-
PLA	18.8	0.5	18.8	-	0.6	-
Metallised film	6.8	0.2	-	6.8	-	0.7
Multilayer PET/PE	6.7	0.2	-	6.7	-	0.7
Unidentified	429.5	10.7	263.6	165.9	8.8	17.1
Sand	32.0	0.8	-	-	-	-
Total	3998.3	100.0	2996.4	969.9	75.5	24.5

**Table 3:** Distribution of PET droplet size (%).

Area (µm^2^)	C3 (wt.%)			
	0	2	5	10
≤25	41	40	35	55
25–50	27	29	34	31
50–75	10	7	17	9
75–100	5	7	7	1
100–125	4	6	1	2
125–150	3	3	2	2
150–175	2	4	1	0
175–200	1	1	0	0
200–225	1	1	0	0
225–250	0	1	1	0
≥250	6	2	1	0
Average area (µm^2^)	70	64	48	32

**Table 4:** Carbonyl index of sMEP and C3-compatibilised blends.

Polybond Content	Carbonyl Index
0%	0.09
2%	0.11
5%	0.26
10%	0.33

**Table 5:** Calorimetric data of the materials.

Material	TmPE (°C)	TmPP (°C)	TmPET (°C)	TcPE (°C)	TcPP (°C)	TcPET (°C)	∆HmPE (J∙g^−1^)	∆HmPP (J∙g^−1^)	∆HmPET (J∙g^−1^)	XcPE (%)	XcPP (%)	XcPET (%)
sMEP	131	165	248	116	-	213	97	5	6	18.3	0.5	1.1
sMEP2%C1	131	164	249	116	-	218	96	5	7	17.6	0.6	1.3
sMEP2%C2	131	165	249	116	-	217	106	5	8	19.5	0.6	1.3
sMEP2%C3	131	163	248	116	-	214	98	5	6	18.0	0.5	1.1
sMEP2%%C123	131	164	249	116	-	216	99	5	7	18.2	0.5	1.2
sMEP5%C1	132	166	249	114	-	213	94	4	6	16.9	0.5	1.0
sMEP5%C2	131	165	249	117	-	217	97	4	7	17.3	0.5	1.2
sMEP5%C3	131	162	249	115	122	216	95	8	7	16.9	0.9	1.3
sMEP5%%C123	131	163	248	116	-	214	99	6	7	17.6	0.7	1.2
sMEP10%C1	131	166	249	114	-	213	103	5	6	17.6	0.6	1.0
sMEP10%C2	131	164	248	117	-	216	94	5	6	16.0	0.6	1.0
sMEP10%C3	131	162	249	116	121	215	86	8	6	14.7	1.1	1.0
sMEP10%C123	131	164	249	115	-	215	91	5	6	15.5	0.7	1.0
sMEP10%C3S1	131	162	248	115	122	211	94	10	6	16.0	1.2	1.0
sMEP10%C3S2	131	162	239	116	122	196	91	9	5	15.2	1.1	3.2
sMEP10%C3S12	131	163	242	116	121	201	93	7	5	15.5	0.9	0.8

## Data Availability

Data are contained within the article and Supplementary Materials.

## Supplementary Materials

The following supporting information can be downloaded at: https://www.mdpi.com/article/10.3390/polym16233441/s1, Figure S1: Average tensile curves of the sMEP blends with 2 wt.%, 5 wt.% and 10 wt.% of C1, C2 and C3 compatibilizers; Figure S2. Average tensile curves of the sMEP blends with 10 wt.% of C3, and S1 and S2 stabilizers.

## References

[1] European Commission (2019). Communication from the Commission to the European Parliament, the European Council, the Council, the European Economic and Social Committee and the Committee of the Regions The European Green Deal. https://eur-lex.europa.eu/legal-content/EN/TXT/?qid=1576150542719&uri=COM%3A2019%3A640%3AFIN.

[2] United Nations (2015). Transforming Our World: The 2030 Agenda for Sustainable Development. https://sdgs.un.org/2030agenda.

[3] DeArmitt C. (2020). The Plastics Paradox: Facts for a Brighter Future.

[4] Berger R. (2023). The Plastic Waste Management Framework.

[5] Kalia V.C., Patel S.K.S., Karthikeyan K.K., Jeya M., Kim I.-W., Lee J.-K. (2024). Manipulating Microbial Cell Morphology for the Sustainable Production of Biopolymers. Polymers.

[6] Zhao X., Korey M., Li K., Copenhaver K., Tekinalp H., Celik S., Kalaitzidou K., Ruan R., Ragauskas A.J., Ozcan S. (2022). Plastic waste upcycling toward a circular economy. Chem. Eng. J..

[7] Ostle C., Thompson R.C., Broughton D., Gregory L., Wootton M., Johns D.G. (2019). The rise in ocean plastics evidenced from a 60-year time series. Nat. Commun..

[8] Parker L. The Great Pacific Garbage Patch Isn’t What You Think It Is. https://education.nationalgeographic.org/resource/great-pacific-garbage-patch-isnt-what-you-think.

[9] Addamo A., Laroche P., Hanke G. (2017). Top Marine Beach Litter Items in Europe.

[10] Araújo M.C.B., Silva-Cavalcanti J.S., Costa M.F. (2018). Anthropogenic Litter on Beaches with Different Levels of Development and Use: A Snapshot of a Coast in Pernambuco (Brazil). Front. Mar. Sci..

[11] Zhang X., Yin Z., Xiang S., Yan H., Tian H. (2024). Degradation of Polymer Materials in the Environment and Its Impact on the Health of Experimental Animals: A Review. Polymers.

[12] Fu Y.-W., Sun W.-F., Wang X. (2020). UV-Initiated Crosslinking Reaction Mechanism and Electrical Breakdown Performance of Crosslinked Polyethylene. Polymers.

[13] Melekhina V.Y., Vlasova A.V., Ilyin S.O. (2023). Asphaltenes from Heavy Crude Oil as Ultraviolet Stabilizers against Polypropylene Aging. Polymers.

[14] Conroy S., Zhang X. (2024). Theoretical insights into chemical recycling of polyethylene terephthalate (PET). Polym. Degrad. Stab..

[15] Galgani F., Hanke G., Maes T., Bergmann M., Gutow L., Klages M. (2015). Global Distribution, Composition and Abundance of Marine Litter. Marine Anthropogenic Litter.

[16] OSPAR (2022). Beach Litter Trend per Survey Site. https://odims.ospar.org/en/submissions/ospar_beach_litter_trend_2022_08/.

[17] Suhaimi N.A.S., Muhamad F., Abd Razak N.A., Zeimaran E. (2022). Recycling of polyethylene terephthalate wastes: A review of technologies, routes, and applications. Polym. Eng. Sci..

[18] Ahmadlouydarab M., Chamkouri M., Chamkouri H. (2020). Compatibilization of immiscible polymer blends (R-PET/PP) by adding PP-g-MA as compatibilizer: Analysis of phase morphology and mechanical properties. Polym. Bull..

[19] Mangold H., von Vacano B. (2022). The Frontier of Plastics Recycling: Rethinking Waste as a Resource for High-Value Applications. Macromol. Chem. Phys..

[20] Dorigato A. (2021). Recycling of polymer blends. Adv. Ind. Eng. Polym. Res..

[21] Dobrovszky K., Ronkay F. (2016). Investigation of compatibilization effects of SEBS-g-MA on polystyrene/polyethylene blend with a novel separation method in melted state. Polym. Bull..

[22] Maris J., Bourdon S., Brossard J.-M., Cauret L., Fontaine L., Montembault V. (2018). Mechanical recycling: Compatibilization of mixed thermoplastic wastes. Polym. Degrad. Stab..

[23] Zhang Y., Zhang H., Guo W., Wu C. (2011). Effects of different types of polyethylene on the morphology and properties of recycled poly(ethylene terephthalate)/polyethylene compatibilized blends. Polym. Adv. Technol..

[24] Araujo L., Morales A. (2018). Compatibilization of recycled polypropylene and recycled poly (ethylene terephthalate) blends with SEBS-g-MA. Polímeros.

[25] Fasce L., Seltzer R., Frontini P., Pita V., Pacheco E., Dias M. (2005). Mechanical and fracture characterization of 50:50 HDPE/PET blends presenting different phase morphologies. Polym. Eng. Sci..

[26] Nomura K., Peng X., Kim H., Jin K. (2020). Multiblock Copolymers for Recycling Polyethylene-Poly(ethylene terephthalate) Mixed Waste. ACS Appl. Mater. Interfaces.

[27] Tang X., Liu C., Keum J., Chen J., Dial B.E., Wang Y., Tsai W.-Y., Bras W., Saito T., Bowland C.C. (2022). Upcycling of semicrystalline polymers by compatibilization: Mechanism and location of compatibilizers. RSC Adv..

[28] Pfaendner R. (2022). Restabilization—30 years of research for quality improvement of recycled plastics Review. Polym. Degrad. Stab..

[29] von Vacano B., Reich O., Huber G., Türkoglu G. (2023). Elucidating pathways of polypropylene chain cleavage and stabilization for multiple loop mechanical recycling. J. Polym. Sci..

[30] (2020). Standard Test Methods for Density and Specific Gravity (Relative Density) of Plastics by Displacement.

[31] (2018). Standard Test Method for Tensile Properties of Thin Plastic Sheeting.

[32] Fávaro S.L., Rubira A.F., Muniz E.C., Radovanovic E. (2007). Surface modification of HDPE, PP, and PET films with KMnO_4_/HCl solutions. Polym. Degrad. Stab..

[33] Fávaro S., Freitas A., Ganzerli T.A., Pereira A.G.B., Cardozo A.L., Baron O., Muniz E., Girotto E.M., Radovanovic E. (2013). PET and aluminum recycling from multilayer food packaging using supercritical ethanol. J. Supercrit. Fluids.

[34] (2021). Standard Test Method for Transition Temperatures and Enthalpies of Fusion and Crystallization of Polymers by Differential Scanning Calorimetry.

[35] Di Lorenzo M.L. (2024). Crystallization of Poly(ethylene terephthalate): A Review. Polymers.

[36] Mehta A., Gaur U., Wunderlich B. (1978). Equilibrium melting parameters of poly(ethylene terephthalate). J. Polym. Sci. Polym. Phys. Ed..

[37] Wang Y., Shi Y., Shao W., Ren Y., Dong W., Zhang F., Liu L.-Z. (2020). Crystallization, Structures, and Properties of Different Polyolefins with Similar Grafting Degree of Maleic Anhydride. Polymers.

[38] Wernet G., Bauer C., Steubing B., Reinhard J., Moreno-Ruiz E., Weidema B. (2016). The ecoinvent database version 3 (part I): Overview and methodology. Int. J. Life Cycle Assess..

[39] Roff W.J., Scott J.R. (2013). Fibres, Films, Plastics and Rubbers: A Handbook of Common Polymers.

[40] de Bomfim A.S.C., Maciel M.M.Á.D., Voorwald H.J.C., Benini K.C.C.d.C., de Oliveira D.M., Cioffi M.O.H. (2019). Effect of different degradation types on properties of plastic waste obtained from espresso coffee capsules. Waste Manag..

[41] Hicks A.L. (2018). Environmental Implications of Consumer Convenience: Coffee as a Case Study. J. Ind. Ecol..

[42] Marinello S., Balugani E., Gamberini R. (2021). Coffee capsule impacts and recovery techniques: A literature review. Packag. Technol. Sci..

[43] Ravati S., Favis B. (2010). Morphological states for a ternary polymer blend demonstrating complete wetting. Polymer.

[44] Yao L., Beatty C., Pourdeyhimi B. (1999). The in situ Compatibilization of HDPE/PET Blends. Imaging and Image Analysis Applications for Plastics.

[45] Chen R.S., Ab Ghani M.H., Salleh M.N., Ahmad S., Gan S. (2014). Influence of Blend Composition and Compatibilizer on Mechanical and Morphological Properties of Recycled HDPE/PET Blends. Mater. Sci. Appl..

[46] Martínez J.G., Benavides R., Guerrero C. (2007). Polyethylenes/PET blend compatibilization with maleic anhydride modified polyethylenes obtained by a UV preirradiation process. J. Appl. Polym. Sci..

[47] Sun J., Pang Y., Yang Y., Zhao J., Xia R., Li Y., Liu Y., Guo H. (2019). Improvement of Rice Husk/HDPE Bio-Composites Interfacial Properties by Silane Coupling Agent and Compatibilizer Complementary Modification. Polymers.

[48] Tarani E., Arvanitidis I., Christofilos D., Bikiaris D.N., Chrissafis K., Vourlias G. (2023). Calculation of the degree of crystallinity of HDPE/GNPs nanocomposites by using various experimental techniques: A comparative study. J. Mater. Sci..

[49] Cestari S.P., Albitres G.A.V., Pires H.M., de França da Silva Freitas D., Mendes L.C. (2019). Study of the Interaction Between Oligomerised Recycled Poly(ethylene terephtalate) and Concrete Waste. J. Polym. Environ..

[50] Lasagabaster A., Abad M.J., Barral L., Ares A. (2006). FTIR study on the nature of water sorbed in polypropylene (PP)/ethylene alcohol vinyl (EVOH) films. Eur. Polym. J..

[51] Caban R. (2022). FTIR-ATR spectroscopic, thermal and microstructural studies on polypropylene-glass fiber composites. J. Mol. Struct..

