# Supplementary material for: Compatibilizer Efficiency in Enhancing Marine Plastic Waste Valorization Through Simulated Recycled Plastic Blends
Source: Polymers (Basel). 2024 Dec 8;16(23):3441. doi: 10.3390/polym16233441 (PMC11644770; doi:10.3390/polym16233441)
Supplement: Supplementary file 1 [file polymers-16-03441-s001.zip › polymers-3311130-supplementary.pdf]

polymers

MDPI

Supplementary material

Compatibilizer Efficiency in Enhancing Marine Plastic Waste Valorization through Simulated Recycled Plastic Blends

Sibele Piedade Cestari[1], Pedro Veiga Rodrigues[1], Ana Cristina Ribeiro[1], Maria Cidália Rodrigues Castro[1], Vasco Cruz[1], Ana Torres[1], Nuno Ramos[1], Ana Vera Machado[1,*]

1 Department of Polymer Engineering, Institute for Polymers and Composites (IPC), Campus de Azurém, University of Minho, 4804-533 Guimarães, Portugal; sibele.cestari@dep.uminho.pt; pedro.rodrigues@dep.uminho.pt; anacmr05@gmail.com; cidaliacastro@dep.uminho.pt; vasco.cruz@dep.uminho.pt; ana.torres@dep.uminho.pt; nuno.ramos@dep.uminho.pt

* Correspondence: avm@dep.uminho.pt;

Supplementary material

Figure S1 shows an example of the tensile curve of each blend, calculated by averaging each tensile curve. Figure S2 shows the tensile curve for blends compatibilized with C3 and stabilizers S1 and S2.

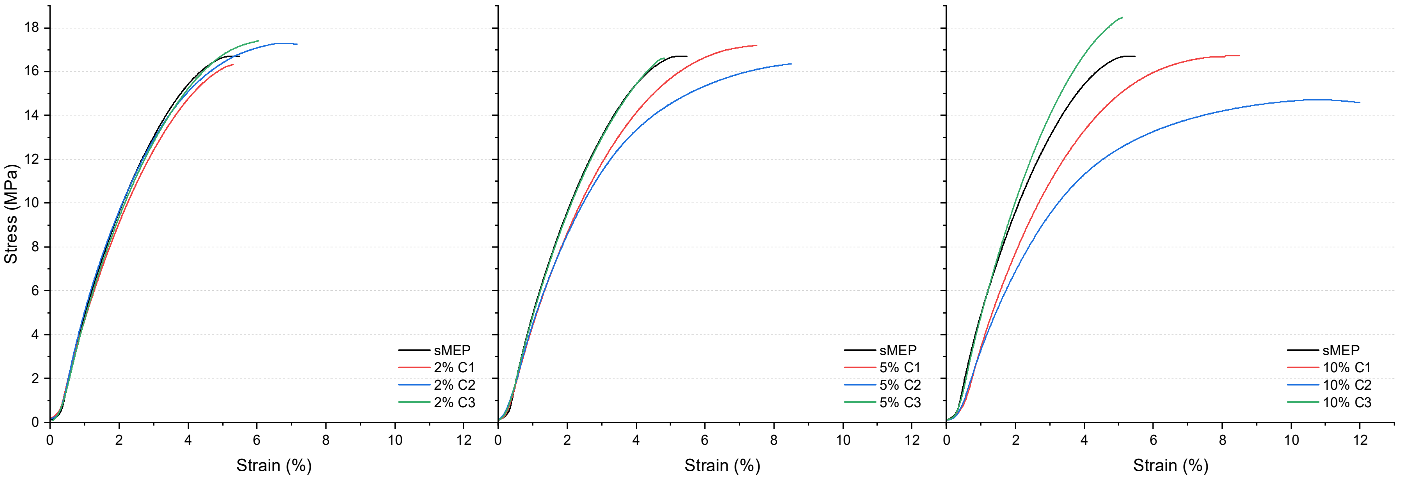

Figure S1. Average tensile curves of the sMEP blends with 2 wt.%, 5 wt.% and 10 wt.% of C1, C2 and C3 compatibilizers.

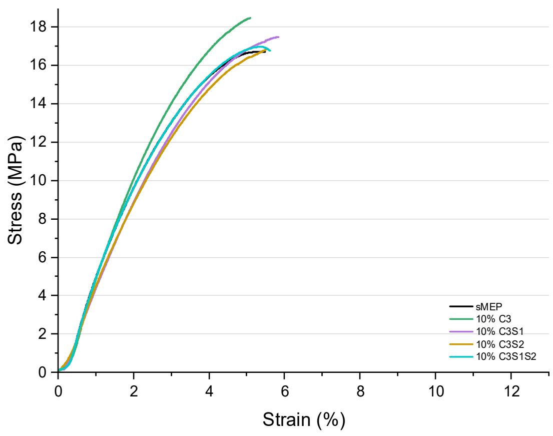

Figure S2. Average tensile curves of the sMEP blends with 10 wt.% of C3, and S1 and S2 stabilizers.

Disclaimer/Publisher's Note: The statements, opinions and data contained in all publications are solely those of the individual author(s) and contributor(s) and not of MDPI and/or the editor(s). MDPI and/or the editor(s) disclaim responsibility for any injury to people or property resulting from any ideas, methods, instructions or products referred to in the content.
